# Movement Disorders and Neuromodulation

**DOI:** 10.1155/2012/309431

**Published:** 2012-09-19

**Authors:** Edward A. Shipton

**Affiliations:** Department of Anaesthesia, University of Otago, P.O. Box 4345, Christchurch 8042, New Zealand

## Abstract

Movement disorders are neurological conditions affecting speed, fluency, quality, and ease of movement. Deep brain stimulation (DBS) is used to treat advanced Parkinson's disease, essential tremor, and dystonia. Possible target sites for DBS include the ventral intermediate nucleus of the thalamus, the globus pallidus internus, and the subthalamic nucleus. High-frequency DBS leads to a kind of functional deafferentation of the stimulated structure and to the modulation of cortical activity. This has a profound effect on the efficiency of movement. Indications for the use of DBS include the need to improve function, reduce medication dependency, and avoid ablative neurosurgery. Appropriate patient selection is critical for success. The implantation technique is briefly described. Programming stimulation parameters are performed via telemetry. The adverse effects of DBS are discussed. The future should see the development of “closed-loop” systems. Its use has promoted interdisciplinary team work and provided an improved understanding of the complex neurocircuitry associated with these disorders. DBS is a highly effective, safe, and reversible surgical treatment for advanced Parkinson's disease, tremor, and dystonia. It is a useful therapeutic option in carefully selected patients that significantly improves motor symptoms, functional status, and quality of life.

## 1. Introduction

Movement disorders are neurological conditions that affect the speed, fluency, quality, and ease of movement. There may be either an excess of movement or a paucity of voluntary and automatic movements, unrelated to weakness or spasticity [1]. Movement disorders can have a profound effect on health and quality of life. Movement is produced and coordinated by several interacting brain structures, such as the motor cortex, the cerebellum, and the basal ganglia (BG) [2]. The motor system is part of the central nervous system that is involved with voluntary and involuntary movements. It consists of the pyramidal and extrapyramidal systems. The extrapyramidal system is part of the motor system that causes involuntary reflexes and movement, and modulation of movement (i.e., coordination). The BG comprises a group of interconnected deep brain nuclei, namely, the caudate and putamen, the globus pallidus internus (GP), the substantia nigra (SN), and the subthalamic nucleus (STN) [2]. These nuclei (via their connections with the thalamus and the cortex) influence the involuntary components of movement and muscle tone. Disruption of such complex circuitry within the BG causes movement disorders, such as Parkinson's disease (PD), essential tremor (ET), and dystonia [2]. The cerebellum contributes to the coordination, precision, and accurate timing of movement. Intimate structural and functional connections between cerebellum and basal ganglia appear to be involved in patients with dystonia [3]. In certain types of dystonia, cerebellar dysfunction (such as compensatory activity) may play a primary role in the pathology of the disorder [3]. Clinical, biochemical, pathological, and imaging studies suggest an abnormal functioning of the cerebellum in ET [4]. 

Movement disorders can be classified as hyperkinesias (excess of movements), dyskinesias (unnatural movements), and abnormal involuntary movements [1]. There is also decreased amplitude of movement (or hypokinesia), but the terms bradykinesia (slowness of movement) and akinesia (loss of movement) are used as well [1]. Movement disorders can develop acutely or over time. For example, acute morbidities encountered in movement disorders include those related to Parkinson's disease, acute drug reactions (acute dystonia, neuroleptic malignant syndrome, serotonergic syndrome, and malignant hyperthermia), acute exacerbation of chronic movement disorders (status dystonicus), hemiballism, and stiff-person syndrome [5]. 

The year 2012 marks the 25th anniversary of the birth of modern DBS. DBS was introduced by Benabid and colleagues in 1987 [6]. It was initially created to treat tremor of the ventral intermediate nucleus (VIM) of the thalamus [7]. Since then, DBS has become a highly effective and safe surgical treatment for severe ET, advanced Parkinson's disease, and dystonia [8]. Its technology is less invasive than stereotactic surgery and is adjustable and reversible. DBS is widely administered with voltage-controlled devices, in which current is variable [9, 10]. High frequency DBS leads to a kind of functional deafferentation of the stimulated structure and to the modulation of cortical activity. This has a profound effect on the efficiency of movement.

Up to date, tens of thousands patients have undergone implantation of DBS electrodes, mainly for the treatment of Parkinson's disease, severe ET, and for primary (idiopathic) dystonia [11]. New uses of DBS include epilepsy and psychiatric disorders such as depression, obsessive compulsive disorder, and Tourette's syndrome [12]. Motor cortex stimulation is used for intractable neuropathic pain (including central poststroke pain). The role of DBS for Parkinson's disease, ET, and dystonia is a well-established treatment option that is currently approved for use in North America, Europe, and in countries such as Australia and New Zealand.

The aims of this narrative paper are to explore the use of DBS in the treatment of movement disorders to review indications for its use and its mechanisms of action. The implantation technique for DBS and its possible adverse effects are examined. Future technological advances clarifying pathophysiology of movement disorders and the need for improved research designs are discussed as well. 

## 2. Methods 

The paper is based on an extensive search of the literature (PubMed, Embase) in relation to the topics covered without strict inclusion or exclusion criteria in the search strategy.

## 3. Indications for Deep Brain Stimulation

Indications for the use of DBS include the need to improve function, reduce medication dependency, and avoid ablative neurosurgery. DBS has arisen to the forefront as a highly effective, safe, and reversible treatment of Parkinson's disease, ET, and dystonia. The possible target sites for DBS include the ventral intermediate (VIM) nucleus of the thalamus, the GPi, and the STN [8]. DBS, especially in the STN, has virtually eliminated ablative surgery.

### 3.1. Parkinson's Disease

Parkinson's disease is a chronic progressive neurodegenerative movement disorder affecting the extrapyramidal motor system. It involves degeneration of the dopaminergic neurones in the SN. The loss of SN pars compacta dopaminergic neurones projecting to the caudate and putamen is considered its neuropathological hallmark [2]. Clinical hallmarks of the condition classically include bradykinesia, rigidity, and resting tremor. Class one evidence exists for the usefulness of DBS for Parkinson's disease [11, 13, 14]. It is estimated that more than 10% of Parkinson's disease patients could benefit from DBS treatment [2]. 

DBS should be reserved for patients with levodopa-responsive Parkinson's disease who have levodopa-related complications that cannot be adequately controlled with medications [15]. The three currently accepted primary targets used for DBS in the treatment of idiopathic advanced Parkinson's disease refractory to medical therapy are the VIM thalamus, the GPi, and the STN [8]. Thalamic DBS primarily relieves tremor with excellent results. STN and GPi DBS alleviate a wide range of Parkinsonian symptoms [8]. The overall clinical outcome of STN and GPi DBS for control of dyskinesia and motor fluctuations is similar [16]. 

Reduction of dopaminergic therapy after STN DBS may help in reducing visual hallucinations and impulse control abnormalities [17]. The use of constant-current bilateral DBS of the STN for Parkinson's disease results in significant improvements in motor function and daily fluctuations of response to levodopa [10]. The evidence to date shows that DBS is generally safe from the cognitive standpoint in well-selected PD patients. However, there is a clear risk of postsurgical cognitive decline that seems greater whenever the STN DBS is used [18]. 

Significant improvements occur in patients with advanced Parkinson's disease (particularly those with severe motor fluctuations) when treated with GPi DBS [7]. These include improvements in gait and posture, reduction of dyskinesias, and the reduction of both the amount and severity of on/off fluctuations [8]. However, both primary and various types of secondary dystonia can be treated very effectively with GPi DBS [8]. 

### 3.2. Tremors

Such tremors include Parkinsonian tremors, ETs, cerebellar tremors, tremors of multiple sclerosis, and orthostatic tremors. 

Parkinsonian tremors are always less responsive to levodopa treatment than the bradykinesia or rigidity [19]. DBS of the VIM thalamus remains an effective target for treatment of certain patients with tremor dominant Parkinson's disease refractory to medical therapy [20]. Contralateral limb tremor is the most improved symptom with thalamic DBS. The frequency of stimulation is a key factor in determining clinical efficacy [21, 22]. Stimulation starts to reduce tremor at a frequency of approximately 50 Hz and reaches a plateau at *∼*200 Hz. For more than five years after implantation, thalamic DBS has been shown to benefit tremor control [20, 23]. In severe Parkinsonian tremor, promising results have recently been obtained from the use of DBS in the posterior subthalamic area (including the caudal zona incerta) [24]. 

ET is the most common movement disorder affecting up to 5.5% of individuals aged 65 years or older [25]. DBS has a profound benefit in ET [26]. The main exclusion criteria of DBS treatment for ET include altered cognition and the presence of an untreated or disabling psychiatric illness [2]. The usefulness of thalamic stimulation in the treatment of essential head and voice tremor remains unproven [8]. 

DBS has been an emerging therapy for disabling cerebellar tremors of different aetiologies (multiple sclerosis, stroke, trauma, cavernous haemangiomas, tumours, and degenerative disease) [8]. DBS of the VIM thalamus reduced tremor in 69% of multiple sclerosis patients [27]. Better control in posttraumatic tremor occurred when dual deep brain stimulator leads were placed over a larger region of the ventral thalamus [8, 28]. Bilateral thalamic stimulation has demonstrated beneficial effects in case reports in treatment-resistant orthostatic tremor [29, 30]. 

### 3.3. Dystonia

Dystonia is the most common type of movement disorder after Parkinson's disease and tremor. It might be primary (idiopathic) or secondary to a known structural lesion of the brain (e.g., cerebral palsy from perinatal hypoxia, infections, stroke, trauma, drugs, and Wilson's disease) or associated with a complex regional pain syndrome [2]. The interaction between the BG and cerebellar circuits plays a major role in its pathophysiology [2]. It presents with sustained, uncontrolled, and often painful muscle contractions causing repetitive movements and abnormal postures [2]. Dystonia is divided into focal (affecting a single body region), segmental (two or more adjacent areas), or generalized (involving the legs, or one leg and the trunk, plus at least one other area of the body) [2]. Focal dystonias include cervical dystonia (spasmodic torticollis), blepharospasm, oculogyric crisis, oromandibular dystonia, spasmodic dysphonia or laryngeal dystonia, and focal hand dystonia [8]. 

The GPi shows abnormal firing activity in dystonia and is therefore the usual target of DBS (e.g., for primary dystonia and for cervical dystonia orspasmodic torticollis) [2]. The optimal frequency and amplitude stimulation settings needed for DBS in dystonia are higher than for GPi DBS and STN DBS in Parkinson's disease patients. The therapeutic effects of GPi DBS for dystonia may take months to occur [31].

Positive effects of DBS on dystonia scales, quality of life, and pain reduction have been confirmed in many studies [2, 32, 33]. In primary generalised dystonia, most studies show improvements of 60–70% on the movement score [34]. Long-term sustainability of these benefits has been demonstrated [35]. In tardive dystonia (from neuroleptics, metoclopramide, and prochlorperazine), there is significant improvement in dystonic symptoms from DBS [36]. 

Whereas the maximum beneficial effect on tremor and rigidity is reached within minutes, the delay for maximal improvement in akinesia is minutes to hours, and the improvement in dystonia gradually develops over several weeks [22, 37–40]. 

## 4. Mechanisms of Deep Brain Stimulation

There are three explanations for the workings of DBS. The first explanation is that high-frequency DBS silences stimulated neurones. The second is that high-frequency stimulation modulates neuronal network activity and neurotransmission [8]. The third is that high-frequency stimulation induces long-term synaptic changes (plasticity).

Recent evidence suggests that DBS has more complex mechanisms of action than the pure functional inactivation of the target region [8]. The ultimate effect of modulating the network activity within the BG can be viewed as the takeover on hyperactive elements or structures of the cortico-BG-thalamocortical complex circuit [8, 41–43]. For example, reducing the abnormally enhanced synchronisation of basal ganglia output is an essential mechanism in the therapeutic effect of DBS in Parkinson's disease. 

Other possible mechanisms of action for high-frequency DBS include local neuronal inhibition with concomitant activation of surrounding fibres, thus resulting in increased synaptic output [43] and activation of afferent axon terminals (e.g., the cortical inputs in the case of high-frequency stimulation of the STN or nucleus accumbens) [22, 44, 45]. This could be of benefit for the treatment of obsessive-compulsive disorders and depression [22, 46]. 

DBS may modulate specific neurones that release specific neurotransmitters, thereby affecting these systems in the brain [8]. The use of volume of tissue-activated studies, other functional imaging, microelectrode multisite recordings, local field potentials, EEGs, and magnetoencephalographic studies will promote understanding of the stimulation effects on local and long-range neuronal networks [6]. 

## 5. Implantation Technique

Appropriate patient selection is critical for success. The selection of candidate patients for DBS should have strict inclusion and exclusion criteria. For example, patient selection criteria for DBS in Parkinson's disease are as follows: (1) a diagnosis of medically refractory intractable Parkinson's disease, primary generalised dystonia, or ET, with symptoms that substantially interfere with the patient's quality of life and functionality, (2) intact cognition, (3) the absence of an untreated or disabling psychiatric illness, (4) realistic expectations, (5) the ability and willingness to participate in regular followup visits, and (6) the absence of comorbidities that are contraindications to DBS [18, 47]. 

**Figure 1:**
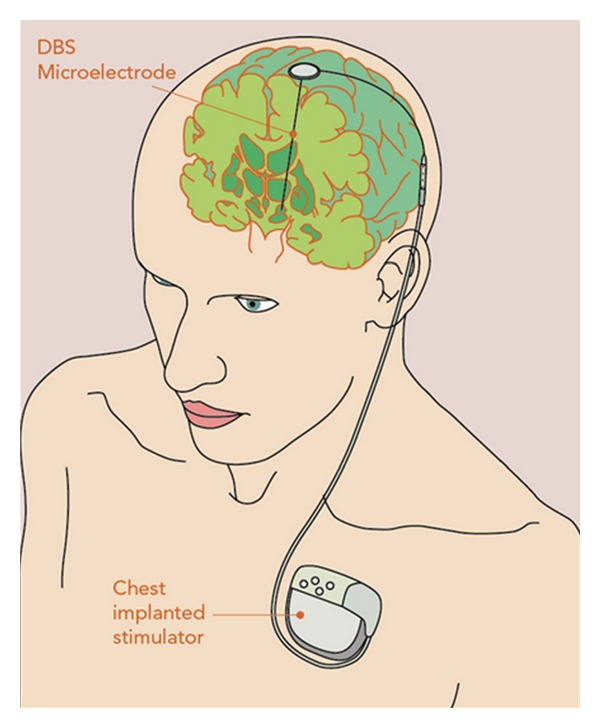
This shows a deep brain stimulator system with the implanted microelectrode; the microelectrode is connected to a programmable stimulator; the stimulator is most often implanted in the subclavicular space.

**Figure 2:**
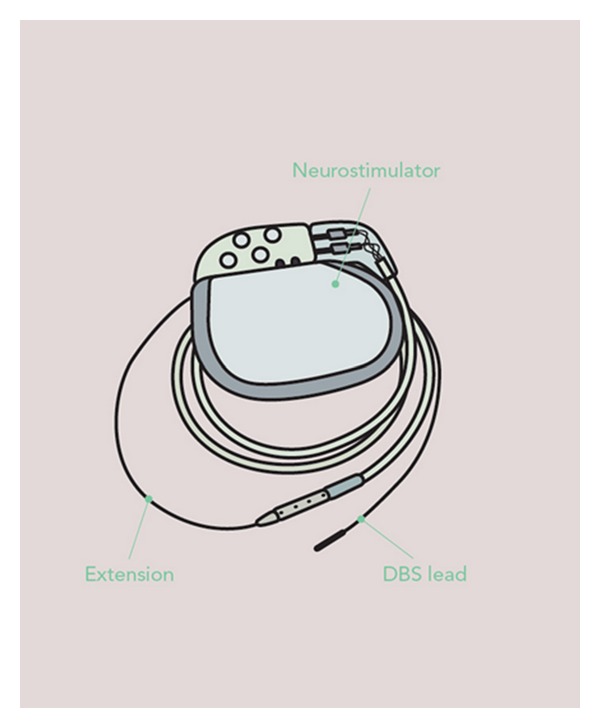
Shows a programmable neurostimulator with a deep brain stimulation lead and extension.

The DBS technique uses continuous high-frequency stimulation of specific brain regions (Figure 1). It involves the implantation of a microelectrode into a deep target within the brain that is connected to a stimulator; the stimulator is programmed to emit electrical impulses at varying strengths and frequencies [8]. Impulses travel to the implanted electrodes from a pulse generator (similar to a cardiac pacemaker) that is telemetrically programmable. Medtronic DBS device (Minneapolis Inc.) is currently the most widely utilised system in functional surgery across the world. The device used has three separate components including the electrode, the extension wires connecting the intracranial electrodes with impulse programming generator (IPG), and the IPG (Figure 2) [48]. The system is programmed and assessed clinically using a hand-held telemetry device.

Although details regarding surgical techniques may vary, all combine a stereotactic technique with detailed image guidance. Stereotaxis is a minimally invasive surgical procedure that makes use of a three-dimensional coordinate system to accurately locate a target in a deep-seated area of the brain. Electrodes are implanted into the target brain area by means of this stereotactic surgical procedure with electrophysiological recordings at the cellular or pathway level [2]. 

A stereotactic head frame is placed on the patient under local anaesthesia in the operating room. A computed tomography (CT) scan or, more commonly, a magnetic resonance imaging (MRI) scan is obtained; this identifies the anterior commissure, posterior commissure, and the midcommissural point [8]. Based on the location of these structures, well-established *x*, *y*, and *z *target coordinates are used to plan electrode placement [8]. Planning software determines the target coordinates; an entry point is found that will allow passage of the electrode through the brain without traversing the ventricle or damaging vascular structures [8]. 

Surgery is usually performed while the patient is awake, off drug therapy, and under local anaesthesia, as this enables reliable microelectrode recording (MER) to be obtained; it allows evaluation of the intraoperative stimulation and possible adverse effects caused by the current diffusion to adjacent structures [49]. General anaesthesia is generally contraindicated during MER, as it depresses neural activity, suppresses clinical symptoms (tremors and rigidity), and interferes with the evaluation of clinical benefits [49]. In patients unable to tolerate an “awake” procedure, ketamine is a safe and effective alternative to other drugs used to induce general anaesthesia, as the feasibility of microelectrode recording is preserved [49]. 

A scalp incision and burr hole are placed in the skull at the predetermined entry point. Electrodes of 1.3 mm in diameter integrating four contacts of 1.5 mm length each, connected to a pulse generator, are used. Microelectrode recording verifies correct electrode placement in deep brain nuclei. Test stimulation is conducted via a temporary external stimulator. Verbal feedback is received from the “awake” patient regarding unwanted adverse effects (such as paraesthesias or visual phenomena). Proper placement is confirmed by intraoperative fluoroscopy and postoperative MRI or CT scanning. 

Once trial stimulation has been deemed successful, a permanent pulse generator (similar to a pacemaker) is placed in the subclavicular space. Stimulation parameters (frequency, amplitude, and pulse widths) may vary. Programming these parameters is performed via telemetry. Several time-consuming visits may be required before the best therapeutic effect is reached.

Bilateral lead implantations can be performed either during a single surgery or in a staged procedure separated by 2–4 weeks. Pulse generators can be placed in a subclavicular position either on the same day or as part of a staged procedure after lead implantation [10]. Successful outcomes are correlated with patient selection, accurate placement of the electrodes in their surgical target, and optimal programming of patients [48]. 

At what stage after the diagnosis of movement disorder should implantation take place? This remains debatable. However, an eight-year followup study in Parkinson's disease showed that STN DBS can be considered safe from a cognitive standpoint but did not seem to modify the cognitive evolution along the course of the disease [50]. On the basis of these observations, it may be appropriate to perform surgery earlier than currently indicated.

## 6. Adverse Effects

### 6.1. Surgical Adverse Effects

Adverse effects noted include those related to the surgery, the hardware, and the stimulation per se. Surgical complications include primarily intracerebral haemorrhage (less than 2% in most centres) and infection (in about 4% of the cases) [2, 51]. Intraoperative or postoperative haemorrhage is the most dreaded complication of DBS [52]. Haemorrhages may occur due to laceration of intracerebral vessels during microelectrode recording or lead implantation. Surgery on the GPi carries a greater haemorrhagic risk than does that on the STN [52]. If infections occur, removal of the hardware is often required. Bleeding and infection can lead to seizures [53]. Reimplantation is performed after an infection clears. 

### 6.2. Hardware Complications

Hardware complications (device-related problems occur in 4.5% of the patients) [54] include the following: erosion over the connector; electrode ruptures or malfunction; electrode migration; lead fractures; infections; skin erosion; battery failure; device malfunction; MRI safety concerns [53]. Erosion of the subcutaneous portions of the hardware occurs in patients with a very low body mass index. Electrode impedance should be checked and recorded at each clinical visit [15, 55]. 

### 6.3. Stimulation-Related Adverse Effects

All patients experience some stimulation-related adverse effects during DBS programming. Stimulation signals with amplitudes greater than those required to achieve symptom control can affect neighbouring structures causing adverse effects; these are reversible with amplitude adjustments [52]. To avoid this, stimulation should be set at amplitudes that do not cause intolerable adverse effects. Dyskinesia, worsening of axial symptoms (freezing, balance, and gait disturbance), speech disturbance, involuntary muscle contractions, paraesthesia, and diplopia are among the common stimulation-related and transient side effects [53]. 

STN DBS can worsen speech and gait in some patients, requiring stimulation parameters to be adjusted. Other adverse effects observed after STN DBS include neuropsychiatric problems, cognitive deterioration, eyelid opening apraxia, weight gain, stimulation-induced dyskinesias, and worsening akinesia [56, 57]. The neuropsychiatric symptoms following STN DBS in Parkinson's disease patients are generally transient and mild if managed appropriately [58]. 

With GPi DBS, adverse effects include paresthesias, muscle contractions, visual flashes, worsening akinesia, dysarthria, weight gain, eyelid opening apraxia, confusion, and cognitive decline [57]. A recent study reported that depression worsened with STN DBS but improved with GPi DBS [59]. 

DBS does not modify the progression of the underlying pathology of Parkinson's disease. Years later, patients can develop disabling levodopa-resistant symptoms, such as gait disturbances and cognitive impairment [2]. Stimulation-induced dyskinesia is frequently managed with a reduction in the dosage of dopaminergic medications. To control symptoms with fewer medication adverse effects, programming of DBS can be performed concurrently with changes in levodopa doses [52]. 

In DBS for ET, the most frequent stimulation-induced adverse effects are paresthesias, followed by dysarthria and pain; these are reversible once the stimulation is turned off. Gait or balance may worsen following DBS for medication refractory ET [60].

### 6.4. Cognitive Effects

Adverse effects of DBS may include modulation of affect, cognition, and behaviour, or possible changes of personality [2]. Some data suggest that the implantation “per se” and not the stimulation is the main cause of the decline in executive function [10]. DBS is generally safe from the cognitive standpoint in well-selected PD patients when looking at measures of global cognition [18]. Nevertheless, there is a clear risk of postsurgical cognitive decline that seems greater whenever the STN DBS is used, although data with other targets is limited [18]. Only one large randomized, double-blind trial has focused mainly on motor efficacy issues of STN DBS versus GPi DBS [59]. Postsurgical decline in verbal fluency has been the most consistently reported cognitive adverse effect in patients undergoing subthalamic DBS [18, 59]. The demonstration of long-term cognitive effects from the surgical procedure or stimulation is difficult. It remains challenging to differentiate these from the natural progression of the disease and other confounding variables (such as drug therapy, brain vascular lesions, PD progression, and concurrent degenerative pathology). Short-term clear cut changes are most probably due to the surgical procedure itself and the electrical stimulation [18]. The factors (such as age, PD duration, disease phenotype, and levodopa responsiveness) that predict postsurgical cognitive decline remain unsatisfactory [18].

## 7. Future

A wireless instantaneous neurotransmitter concentration system (WINCS) has been developed to promote understanding of the neurocircuitry involved [61, 62]. The WINCS system provides real-time neurotransmitter monitoring to reveal underlying neuromodulatory mechanisms of DBS action [63]. This device is capable of monitoring the release of a variety of neurochemicals (dopamine, serotonin, histamine, and adenosine) during DBS using the electroanalytical techniques of fast-scan cyclic voltammetry at a carbon fibre microelectrode, and fixed potential amperometry [64] at enzyme-linked biosensors [8]. 

The future should see the development of “closed-loop” DBS systems; these would provide feedback from brain electrical activity to direct the stimulation and neuroimaging modalities. Computational analysis or electrophysiological modelling can be used to enhance DBS [65]. DBS would then depend on the use of multiple electrodes with these “closed-loop” systems that include macrorecordings and stimulation [2]. It might even allow the performance of effective and safe programming through remote access, such as via the telephone or the internet [2]. By disentangling the neuronal network codes, closed-loop devices [66] could provide stimulation “on demand” [11]. 

Closed-loop DBS is currently in clinical trials for refractory epilepsy [65]. On-going clinical trials with DBS are investigating its use in tremor in multiple sclerosis, in mood disorders, in pain and cluster headache, in hypertension, in obesity, in memory impairment, in aggressiveness, in drug addiction, and in other central nervous system disorders; this will enhance indications for its use in future.

Advances in functional imaging are providing “new” brain targets for an increasing number of pathologies [7]. New imaging techniques offer preoperative modelling for DBS surgery, including nerve fibre tracts (diffusion tensor imaging), and imaging of volume of tissue activated by a specific electrode [65]. Computational analysis techniques for DBS include mathematical models of the abnormally synchronized electrical activity that underlies epilepsy, movement disorders, and many mood disorders as well.

New programming options such as interleaving [67] and constant current devices [10] are now on the market. Constant-current stimulation provides more accurate control of the spread of the electrical field than do voltage-controlled devices, as adjustments can be for heterogeneity in tissue impedance [10, 68]. Future trials should compare constant-current with voltage-controlled stimulation. The development of new electrodes with improved variability of stimulation direction should aid progress as well. DBS technology in future will consist of multicontact electrodes and sensing capabilities.

Finding the right anatomical areas to stimulate to gain the best outcomes remains a challenge. A more recent experimental target is the pedunculopontine nucleus (PPN) that may be appropriate for patients with gait freezing or postural instability gait difficulty [69–71]. The centremedian/parafascicular thalamic complex has been proposed as a successful target for control of tremor as well [71]. 

Fibre tracts rather than nuclei might be the correct target of choice (not only in Parkinson's disease, but also in thalamic stimulation for ET). Optogenetic studies suggest that STN stimulation and stimulation of afferents from cortical areas might form the main mechanism of action of DBS [11, 41]. 

## 8. Conclusion

Movement disorders encompass acute and chronic diseases characterised by involuntary movements or loss of control or efficiency in voluntary movements [5]. In movement disorders, DBS is a highly effective, safe, and reversible surgical treatment for advanced Parkinson's disease, tremor, and dystonia. Its use has promoted interdisciplinary clinical team work and provided an improved understanding of the complex neurocircuitry associated with these disorders. For improvement of outcomes after DBS, a refinement of patient selection criteria is needed [71]. DBS is a useful therapeutic option in carefully selected patients that significantly improves motor symptoms, functional status, and quality of life [72]. DBS remains an expensive resource, and its future clinical use will continue to raise many regulatory and ethical issues. Further evidence, particularly in the form of prospective studies and randomised controlled trials, is required to better establish the pathophysiology of movement disorders and its role therein.
